# THE INFLUENCE OF SOCIAL ISOLATION AND NEIGHBORHOOD DANGER ON OLDER ADULTS’ FUNCTIONAL STATUS

**DOI:** 10.1093/geroni/igz038.1947

**Published:** 2019-11-08

**Authors:** Ilana J Engel, Tamara A Baker

**Affiliations:** University of Kansas, Lawrence, Kansas, United States

## Abstract

Social isolation is often associated with smaller social networks, bereavement, and chronic health problems. In addition, underserved neighborhoods, without the resources and social support of other areas, may further promote social isolation among older adults. This study utilized data from the 2nd wave of the nationally representative National Social Life, Health, and Aging Project (NSHAP) to examine if perceived neighborhood danger mediates the relationship between social isolation and functional impairment. We hypothesized that those who are less socially connected and feel less safe in their communities may experience worse health outcomes. Data for the total sample (N=1,804; 62-91 years of age) showed that partial mediation was supported, (F 2, 1801 = 22.91, p<0.01). Similar statistics were found by gender (men, F 2, 985 = 8.20, p<0.01; women, F 2, 813 = 14.79, p<0.01). This relationship, however, showed a stronger association among women (β = -.39, p<.01) than men (β = -.26, p<.05). Findings indicate that the relationship between perceived social isolation and impaired functional status may be partially explained by perceived neighborhood danger. These findings suggest that older adults who perceive their neighborhoods as dangerous, may be more socially isolated, and at risk for functional decline. These results support the Reserve Capacity Model, which posits that social resources are of increased importance for socioeconomically disadvantaged individuals. Additional research is needed to examine how such factors as stress, environment, and access to care contribute to our understanding of health outcomes among this population of adults.

